# Ovariectomy attenuates phenotypes related to Alzheimer’s disease in a preclinical mouse model and in C57BL/6 J mice

**DOI:** 10.1038/s41598-025-20006-9

**Published:** 2025-10-28

**Authors:** Kazuki Fujii, Yumie Koshidaka, Yuko Yanagibashi, Mayumi Adachi, Mina Matsuo, Kimihiro Kimura, Takashi Saito, Takaomi C. Saido, Keizo Takao

**Affiliations:** 1 Department of Behavioral Physiology, Faculty of Medicine, University of Toyama, 2630 Sugitan, Toyama, 930-0194 Japan; 2 Life Science Research Center, University of Toyama, Toyama, Japan; 3 Department of Behavioral Physiology, Graduate School of Innovative Life Science, University of Toyama, Toyama, Japan; 4 Research Center for Idling Brain Science, University of Toyama, Toyama, Japan; 5 Department of Neurocognitive Science, Institute of Brain Science, Nagoya City University Graduate School of Medical Sciences, Nagoya, Japan; 6 Laboratory for Proteolytic Neuroscience, RIKEN Center for Brain Science, Wako, Saitama Japan

**Keywords:** Alzheimer's disease, Cognitive ageing

## Abstract

Women are at higher risk for Alzheimer’s disease (AD) than men and hormonal changes during perimenopause are considered a risk factor. The relationship between ovarian hormones and AD has been explored using AD animal models, especially through ovariectomy (OVX) in established AD models. The link between early-stage AD and ovarian hormones, however, remains unclear, largely due to the lack of suitable animal models. Appropriate models for studying early-stage AD pathology, treatment, and prevention are critically needed. The *App* knock-in mouse model, which carries a single amyloid precursor protein (*App*) gene mutation, effectively reproduces early amyloid AD pathology. To elucidate the relationship between ovarian hormone deficiency and the behavioral phenotypes of a preclinical AD model, we applied a comprehensive behavioral test battery to this mouse model with bilateral OVX. The *App* mutation reduced anxiety-like behavior and impaired performance in the fear memory task. OVX restored the anxiety-like behavior of the *App* mutation mice to a level comparable to that in wild-type (WT) mice. Furthermore, OVX enhanced performance in a fear memory task in both genotypes and reduced amyloid-β staining in WT mice. Together, these findings suggest that OVX attenuates AD-related phenotypes in a preclinical AD model and in C57BL/6 J WT mice.

## Introduction

Alzheimer’s disease (AD) is a neurodegenerative disorder characterized by the progressive deposition of extracellular amyloid-β protein and cognitive impairment. In the United States, approximately 10% of the population aged ≥ 65 has dementia caused by AD^1^. In today’s aging society, AD is a major concern, and treatments to prevent or delay the onset of AD are critically needed. Clinical studies have demonstrated that women are at higher risk for AD than men^2–4^. Hormonal changes during menopause are considered a risk factor for AD^5^. While replacement hormone therapy might protect postmenopausal females against cognitive decline and AD^6^, some reports suggest that hormonal therapy increases the risk of dementia^7,8^. The clinical benefits of hormonal therapy are considered to outweigh the risks^9^, although the link between ovarian hormones and AD pathology remains unclear.

To develop effective treatments for AD, animal models replicating the main phenotypic characteristics of human AD are needed. Over the past several decades, more than 100 animal models have been developed and used to advance biomedical research in AD^10,11^. Many of these models are transgenic mice that overexpress the human amyloid precursor protein (*App*) gene, based on the amyloid hypothesis^12^. These models display pathologic and behavioral AD-related phenotypes, such as amyloid plaques, tau aggregation, and memory impairment^10,13^. To explore sex-specific and menopause-related AD pathologies, the effects of sex hormones have also been studied in animal models of AD^14–22^. In particular, ovariectomy (OVX) models are widely used and play an important role in elucidating the link between sex steroids and AD pathology^23–27^. A recent report, however, suggested that some AD models may not actually replicate AD pathology because they are based on App or Tau overexpression^13^. To address this issue, we developed single *App* knock-in model mice (*App*^*NL-G-F/NL-G-F*^ mice), which carry a single amyloid precursor protein (*App*) gene mutation, effectively reproducing the pathologic and behavioral phenotypes of preclinical AD pathology^28,29^. In such a preclinical AD model, however, it remains unclear how ovarian hormonal changes, particularly those associated with the perimenopausal and postmenopausal periods, affect both the pathologic features and behavioral phenotype associated with AD. Here, we conducted a comprehensive behavioral test battery to investigate the effect of OVX on the behavior of wild-type (WT) and preclinical AD model mice. Our findings revealed that OVX attenuated the AD-like phenotypes not only in preclinical AD model mice but also in WT mice.

## Results

### ***OVX induced weight gain in WT mice, but not in App***^***NL-G-F/NL-G-F***^*** mice***

**Fig. 1:**
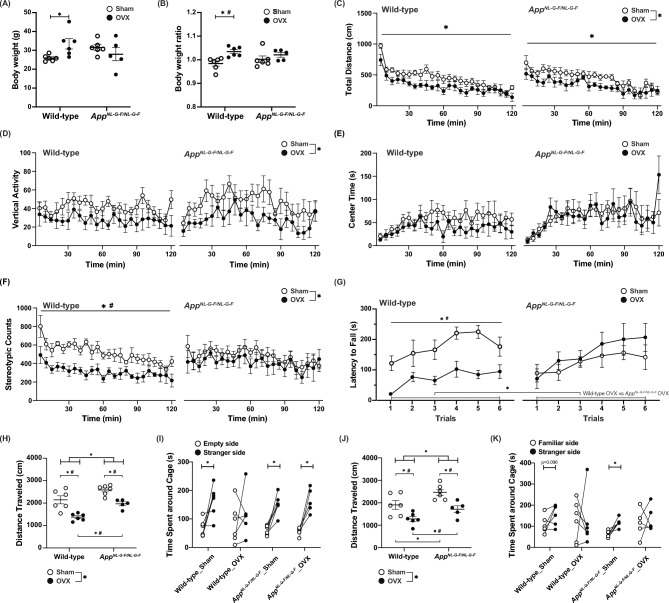
*App*^*NL-G-F/NL-G-F*^ mice exhibited reduced susceptibility to ovariectomy (OVX)-induced weight gain and some behavioral alterations. Body weight at 35 weeks of age (**A**) and body weight gain ratio from 35 to 37 weeks of age (**B**) are shown. OVX induced weight gain in wild-type (WT) mice. Data are presented as dot plot individual values and means ± S.E.M. (Sham, n = 6/WT or 6/*App*^*NL-G-F/NL-G-*F^; OVX, n = 6/WT or 5/*App*^*NL-G-F/NL-G-F*^). Open field test results of OVX WT and App^NL-G-F/NL-G-F^ mice. Distance traveled (**C**), vertical activity (**D**), time spent in the center of the compartment (**E**), and stereotypic counts (**F**) every 5 min for 120 min are shown. Data are presented as means ± S.E.M. for the indicated numbers of animals. In the rotarod test, the latency to fall from the accelerating rotarod was measured in three trials per day (**G**). OVX WT mice showed a shorter latency to fall. Data are presented as means ± S.E.M. for the indicated numbers of animals (Sham, n = 6/WT or 6/*App*^*NL-G-F/NL-G-F*^; OVX, n = 6/WT or 5/*App*^*NL-G-F/NL-G-F*^). In the sociability and social novelty preference test, the total distance traveled of each test (**H, J**), the time spent around the cage with stranger mice or around the empty cage (**I**) and time spent around the cages with stranger mice and familiar mice (**K**) are shown. Data are presented as means ± S.E.M. for the indicated numbers of animals (Sham, n = 6/WT or 6/*App*^*NL-G-F/NL-G-F*^; OVX, n = 6/WT or 5/*App*^*NL-G-F/NL-G-F*^). One‐way analysis of variance (**H, J**) and paired t-test (**I,K**) were used to test for statistical significance. The asterisk (*) indicates a significant difference (*p* < 0.05) and the number sign (#) indicates significance after Bonferroni correction for multiple comparisons, with the adjusted threshold set at *p* < 0.0125 (0.05/4).

To evaluate the effects of OVX on the behavioral characteristics of WT and single *App* knock-in AD model mice, we ovariectomized or sham-operated *App*^*NL-G-F/NL-G-F*^ mice and their WT littermates at 18 weeks of age. From 35 weeks of age, the mice were subjected to a comprehensive battery of behavioral tests (Table 1). The neurological screen and neuromuscular strength test showed a significant interaction between genotype and OVX on body weight (treatment effect, *p* = 0.3827; genotype effect, *p* = 0.9789; treatment × genotype, *p* = 0.0221; Fig. 1A). Body weight was higher in OVX WT mice than in sham-operated WT mice, while body weight was similar between OVX *App*^*NL-G-F/NL-G-F*^ mice and sham-operated *App*^*NL-G-F/NL-G-F*^ mice (sham-operated WT vs OVX WT, *p* = 0.0240; sham-operated *App*^*NL-G-F/NL-G-F*^ vs OVX *App*^*NL-G-F/NL-G-F*^, *p* = 0.2835; Fig. 1A). The interaction between genotype and OVX treatment on body weight remained significant at 37 weeks of age (treatment effect, *p* = 0.6536; genotype effect, *p* = 0.9699; treatment × genotype, *p* = 0.0236; Table 2). When comparing body weight gain from 35 to 37 weeks of age among groups, OVX WT mice showed a significantly greater gain than sham-operated WT mice, whereas *App*^*NL-G-F/NL-G-F*^ mice showed no significant differences between OVX and sham-operated mice (Kruskal–Wallis test, *p* = 0.0239; sham-operated WT vs OVX WT, *p* = 0.0039; sham-operated *App*^*NL-G-F/NL-G-F*^ vs OVX *App*^*NL-G-F/NL-G-F*^, *p* = 0.2652; Fig. 1B). These findings suggest that *App*^*NL-G-F/NL-G-F*^ mice are resistant to OVX-induced weight gain.

**Table 1:** Comprehensive behavioral test battery schedule of ovariectomized wild-type and *App*^*NL-G-F/NL-G-F*^ mice.

Order	Test	Age (w)	Table/Figure
1	Neurological screens and neuromuscular strength test (GHNS)	35–36	Figure 1
2	Light/dark transition test (LD)	36	Figure 2
3	Open field test (OF)	36	Figure 1
4	Hot plate test (HP)	36	Table2
5	Social interaction test (SI)	37	Table2
6	Elevated plus maze test (EP)	37	Figure 2
7	Rotarod test (RR)	37–38	Figure 1
8	Startle response/prepulse inhibition test (PPI)	38	Table2
9	Y-maze test (YM)	38	Figure 3
10	T-maze test (TM)	42	Figure 3
11	Three-chamber social approach test (TCSI)	44	Figure 1
12	Contextual and cued fear conditioning test (FZ)	46–50	Figure 4

**Table 2:** Statistical analyses of behavioral data.

Test	Measure	Figure	Means ± SEM				P-value					
			Wild-type_Sham	Wild-type_OVX	*App*^*NL-G-F/NL-G-F*^_Sham	*App*^*NL-G-F/NL-G-F*^_OVX	Genotype	OVX	Genotype × OVX	Genotype × Time/Trial	OVX × Time/Trial	Genotype x OVX × Time/Trial
Neurological screens and neuromuscular strength test	Body weight (g)	Figure1	25.898 ± 0.668	33.473 ± 2.732	31.533 ± 1.479	27.958 ± 3.47	p = 0.9789	p = 0.3827	p = 0.0221	-	-	-
	Body temperature (˚C)	-	36.283 ± 0.263	36.25 ± 0.368	36.533 ± 0.22	36.4 ± 0.838	p = 0.6591	p = 0.8538	p = 0.912	-	-	-
	Grip strength (N)		0.542 ± 0.064	0.525 ± 0.041	0.521 ± 0.051	0.485 ± 0.036	p = 0.5551	p = 0.6101	p = 0.8519	-	-	-
	Wire hang (s)		43.975 ± 10.138	30.522 ± 9.004	33.63 ± 8.303	32.444 ± 11.707	p = 0.6707	p = 0.4621	p = 0.5368	-	-	-
Light/dark transition test	Distance traveled (cm) in light chamber	Figure2	619 ± 87.462	1572.783 ± 29.43	628.05 ± 76.052	387.44 ± 46.627	p = 0.0634	p = 0.0013	p = 0.1948	p = 0.0278	p = 0.8196	p = 0.1917
	Distance traveled (cm) in dark chamber		1640.967 ± 50.523	1572.783 ± 42.513	1560.767 ± 74.963	1222.74 ± 129.349						
	Transitions		24.5 ± 2.566	18.167 ± 0.98	26.5 ± 3.202	15.4 ± 1.887	p = 0.8727	p = 0.0015	p = 0.3254	-	-	-
	Latency to light chamber (s)		56.833 ± 16.481	45.333 ± 10.468	57.333 ± 9.401	86.4 ± 34.916	p = 0.2833	p = 0.6461	p = 0.2947	-	-	-
	Time spent in light chamber (s)		147.667 ± 13.89	104.917 ± 5.037	161.25 ± 16.899	117.8 ± 15.954	p = 0.3442	p = 0.0052	p = 0.9798	-	-	-
Open field test	Distance traveled (cm)	Figure1	10724.5 ± 834.12	7621.33 ± 834.52	10860.33 ± 740.1	7942.4 ± 746.78	p = 0.7781	p = 0.0013	p = 0.909	p = 0.0115	p = 0.358	p = 0.9604
	Vertical activity		988.5 ± 105.837	661.5 ± 126.865	1105.5 ± 159.538	677.6 ± 161.897	p = 0.6383	p = 0.0139	p = 0.7212	p = 0.1423	p = 0.8104	p = 0.8649
	Center time (s)		1354.1167 ± 223.69	899.37 ± 202.98	1551.78 ± 336.19	1525.32 ± 328.72	p = 0.1525	p = 0.3947	p = 0.4479	p = 0.0912	p = 0.554	p = 0.4712
	Stereotypic counts		12380.67 ± 975.831	7430.83 ± 834.70	11512.5 ± 1354.9127	9810.8 ± 1838.923	p = 0.5554	p = 0.0161	p = 0.2126	p = 0.0031	p = 0.3104	p = 0.7432
Hot plate test	Hot plate latency (s)	-	5.83 ± 0.856	5.565 ± 0.662	4.653 ± 0.549	5.23 ± 0.997	p = 0.3374	p = 0.8414	p = 0.5901	-	-	-
	Body weight (g)		26.348 ± 0.825	32.273 ± 2.396	31.425 ± 1.182	27.352 ± 3.267	p = 0.9699	p = 0.6536	p = 0.0236	-	-	-
Social interaction test	Distance traveled (cm)	-	3608.367 ± 91.623	3176.867 ± 182.795	4045.033 ± 115.13	3186.6 ± 190.4	p = 0.1725	p = 0.0032	p = 0.1895	-	-	-
	Number of contacts		55 ± 6.658	54 ± 12.741	57.333 ± 6.173	57.5 ± 10.5	p = 0.7681	p = 0.9663	p = 0.9528	-	-	-
	Total duration of contacts (s)		65.867 ± 10.785	74.667 ± 20.651	68.1 ± 7.351	66.15 ± 12.15	p = 0.8336	p = 0.8189	p = 0.7202	-	-	-
	Total duration of active contacts (s)		16.233 ± 1.576	16.533 ± 3.762	17.433 ± 2.426	18.7 ± 4	p = 0.5913	p = 0.801	p = 0.8762	-	-	-
	Mean duration of contact (s)		1.2 ± 0.115	1.367 ± 0.067	1.2 ± 0.115	1.15 ± 0.05	p = 0.3205	p = 0.5829	p = 0.3205	-	-	-
Elevated plus maze test	Distance traveled (cm)	Figure2	1715.367 ± 74.345	1475.067 ± 172.602	1949.033 ± 79.646	1260.36 ± 253.294	p = 0.9512	p = 0.0067	p = 0.1585	-	-	-
	Number of entries		36.667 ± 1.892	31.667 ± 4.169	45.167 ± 3.07	28.8 ± 7.038	p = 0.5091	p = 0.0195	p = 0.1904	-	-	-
	Entries into open arms (%)		20.433 ± 1.812	24.217 ± 3.02	36.917 ± 4.453	25.16 ± 1.721	p = 0.0106	p = 0.2103	p = 0.0205	-	-	-
	Time on open arms (%)		2.383 ± 0.261	3.167 ± 0.658	15.25 ± 1.436	4.14 ± 1.852	p <0.0001	p = 0.0003	p <0.0001	-	-	-
Rotarod test	Latency to fall (s) of 1 st trial	Figure1	121.667 ± 24.09	21 ± 4.531	89.333 ± 28.591	71.2 ± 32.234	p = 0.5486	p = 0.1384	p = 0.012	p = 0.6254	p = 0.3691	p = 0.4042
	Latency to fall (s) of 2nd trial		154.667 ± 43.174	76.667 ± 15.385	92 ± 26.327	130 ± 29.04						
	Latency to fall (s) of 3rd trial		165.833 ± 32.194	65.333 ± 14.141	128.167 ± 14.849	136 ± 27.384						
	Latency to fall (s) of 4th trial		220.667 ± 19.852	102.5 ± 26.093	146.5 ± 38.167	185.6 ± 38.296						
	Latency to fall (s) of 5th trial		225.167 ± 21.327	84.667 ± 13.61	156.333 ± 23.056	200.6 ± 52.211						
	Latency to fall (s) of 6th trial		176.333 ± 31.282	94.5 ± 22.39	141.5 ± 40.626	206.8 ± 45.801						
Startle response test	Startle response (110 dB)	-	0.315 ± 0.081	0.125 ± 0.008	0.298 ± 0.101	0.468 ± 0.164	p = 0.1722	p = 0.9221	p = 0.1317	p = 0.6776	p = 0.9606	p = 0.6286
	Startle response (120 dB)		0.418 ± 0.156	0.163 ± 0.034	0.393 ± 0.27	0.616 ± 0.177						
Prepulse inhibition test	PPI (74-110 dB)		46.279 ± 12.964	44.982 ± 6.422	50.943 ± 6.121	21.892 ± 32.359	p = 0.8586	p = 0.4944	p = 0.6741	p = 0.1435	p = 0.1889	p = 0.0797
	PPI (78-110 dB)		53.776 ± 10.08	48.077 ± 4.535	55.509 ± 7.054	55.401 ± 19.288						
	PPI (74-120 dB)		30.725 ± 17.051	24.738 ± 12.702	5.847 ± 19.095	50.328 ± 13.396	p = 0.683	p = 0.118	p = 0.0864	p = 0.2869	p = 0.3854	p = 0.7531
	PPI (78-120 dB)		51.092 ± 15.8	51.947 ± 5.209	9.213 ± 25.492	68.206 ± 6.734						
Three-chamber social approach test (Sociability test)	Distance traveled (cm)		2144.933 ± 179.249	1375.867 ± 64.832	2585.417 ± 90.836	1969.02 ± 88.026	p = 0.0003	p <0.0001	p = 0.5228			
		Figure1								-	-	-
	The ratio of time spent around cage		0.695 ± 0.063	0.605 ± 0.082	0.713 ± 0.03	0.74 ± 0.039	p = 0.7678 (Kruskal-Wallis Test)			-	-	-
Three-chamber social approach test (Social novelty preference test)	Distance traveled (cm)		1907.75 ± 196.066	1282.183 ± 110.3	2467.95 ± 137.499	1715.32 ± 151.916	p = 0.0043	p = 0.0002	p = 0.6829	-	-	-
	The ratio of time spent around cage		0.59 ± 0.046	0.493 ± 0.113	0.649 ± 0.034	0.559 ± 0.094	p = 0.391 (Kruskal-Wallis Test)			-	-	-
Y-maze test	Number of entries	Figure3	23.333 ± 1.978	18.5 ± 1.708	23.5 ± 2.012	13.2 ± 3.412	p = 0.2721	p = 0.0035	p = 0.2432	-	-	-
	Total alternation		11.5 ± 2.029	7.167 ± 1.222	11 ± 1.317	5 ± 1.975	p = 0.4311	p = 0.0057	p = 0.6209	-	-	-
	Alternation (%)		69.633 ± 5.138	65.55 ± 9.735	64.767 ± 4.778	43.76 ± 11.746	p = 0.1153	p = 0.1368	p = 0.3078	-	-	-
	Total distance (cm)		2152.967 ± 192.856	1761.783 ± 213.469	2393.883 ± 152.075	1599.28 ± 324.587	p = 0.8608	p = 0.0146	p = 0.372			
T-maze test	Correct responses(%)	Figure3	67 ± 3.416	71 ± 2.863	67 ± 2.463	68.532 ± 3.744	p = 0.6957	p = 0.3878	p = 0.6957	p = 0.7459	p = 0.9689	p = 0.919
	Latency		878.917 ± 146.007	1232.083 ± 128.815	716.333 ± 66.585	935.5 ± 153.909	p = 0.1956	p = 0.0097	p = 0.6126	p = 0.0023	p = 0.747	p = 0.0034
	Distance (cm)		2706.13 ± 160.714	2716.23 ± 81.885	2573.973 ± 75.559	2749.47 ± 79.976	p = 0.6533	p = 0.4025	p = 0.4548	p = 0.2615	p = 0.7453	p = 0.0143
Fear conditioning test (Conditioning)	Freezing (%)	Figure4	40.48 ± 4.036	51.22 ± 4.96	39.27 ± 5.55	48.25 ± 8.81	p = 0.7245	p = 0.1078	p = 0.8817	p = 0.0016	p = 0.0722	p = 0.1716
Fear conditioning test (Context test 1 day after conditioning)	Freezing (%)		42.28 ± 7.26	56.66 ± 5.15	38.34 ± 3.52	56.27 ± 8.25	p = 0.7285	p = 0.0166	p = 0.7764	p = 0.4747	p = 0.8376	p = 0.8896
Fear conditioning test (Cued test 1 day after conditioning (pre-CS))	Freezing (%)		11.48 ± 2.79	25.09 ± 4.9	17.39 ± 5.88	27.57 ± 6.81	p = 0.4288	p = 0.0334	p = 0.7438	p = 0.1767	p = 0.7346	p = 0.7452
Fear conditioning test (Cued test 1 day after conditioning (CS))	Freezing (%)		42.58 ± 4.98	66.85 ± 4.67	34.35 ± 8.26	50.77 ± 12.4	p = 0.1338	p = 0.0168	p = 0.6191	p = 0.1631	p = 0.7147	p = 0.2043
Fear conditioning test (Context test 30 days after conditioning)	Freezing (%)		42.73 ± 5.77	71.44 ± 5.46	39.28 ± 4.27	53.33 ± 6.67	p = 0.0665	p = 0.001	p = 0.2012	p = 0.8836	p = 0.5571	p = 0.4141
Fear conditioning test (Cued test 30 days after conditioning (pre-CS))	Freezing (%)		36.21 ± 4.19	44.35 ± 6.02	34.63 ± 6.40	48.68 ± 6.53	p = 0.8158	p = 0.0726	p = 0.619	p = 0.6119	p = 0.3185	p = 0.7947
Fear conditioning test (Cued test 30 days after conditioning (CS))	Freezing (%)		53.06 ± 5.97	74.92 ± 4.66	29.62 ± 6.90	59.11 ± 9.16	p = 0.0084	p = 0.0011	p = 0.5741	p = 0.0025	p = 0.9325	p = 0.169
Fear conditioning test (footshock)	Distance traveled (cm)	-	288.25 ± 11.17	278.35 ± 16.86	301.35 ± 10.03	267.54 ± 13.4	p = 0.9317	p = 0.1138	p = 0.3759	p = 0.1359	p = 0.9999	p = 0.7176

### ***OVX reduced stereotypic behavior and impaired motor coordination in WT mice, but not in App***^***NL-G-F/NL-G-F***^*** mice***

A similar pattern was observed in other experiments as well. In the open field test, OVX significantly reduced locomotor activity and vertical activity (Fig. 1C, D) in both genotypes. OVX also significantly affected stereotypic counts; the increase in stereotypic counts, however, was significant only in WT mice. No significant differences in the time spent in the center area were detected among the groups (Fig. 1E). While the stereotypic count was lower in OVX WT mice than in sham-operated WT mice, no significant differences were detected between sham-operated and OVX *App*^*NL-G-F/NL-G-F*^ mice (Fig. 1F).

We performed a rotarod test to investigate how OVX and the *App* mutation affect motor coordination in mice. The latency to fall was significantly shorter in OVX WT mice than in WT sham-operated controls and OVX *App*^*NL-G-F/NL-G-F*^ mice (treatment effect, *p* = 0.1384; genotype effect, *p* = 0.5486; treatment × genotype, *p* = 0.0121; treatment × time, *p* = 0.3691; genotype × time, *p* = 0.6254; treatment × genotype × time, *p* = 0.4042; sham-operated WT vs OVX WT, *p* = 0.0017; OVX WT vs OVX *App*^*NL-G-F/NL-G-F*^, *p* = 0.0361; Fig. 1G). In contrast, no significant difference in the latency to fall was detected between OVX *App*^*NL-G-F/NL-G-F*^ mice and sham-operated controls (sham-operated *App*^*NL-G-F/NL-G-F*^ vs OVX *App*^*NL-G-F/NL-G-F*^*, p* = 0.5061, Fig. 1G). Therefore, OVX induced motor coordination impairments in WT mice. Although baseline motor coordination performance tended to be poorer in *App*^*NL-G-F/NL-G-F*^ mice than in WT mice, OVX treatment did not affect motor coordination in *App*^*NL-G-F/NL-G-F*^ mice. In addition, OVX *App*^*NL-G-F/NL-G-F*^ mice exhibited better motor coordination performance than OVX WT mice. These results suggest that OVX differentially affects motor coordination depending on the genotype.

### ***OVX induced sociability deficits in WT mice, but not in App***^***NL-G-F/NL-G-F***^*** mice***

We also examined the social behavior of OVX *App*^*NL-G-F/NL-G-F*^ and WT mice using the social interaction test in a novel environment and the three‐chamber social approach test. In the social interaction test conducted in a novel environment, OVX decreased locomotor activity in both genotypes, while no significant differences in other parameters—including social contacts—were detected among groups (Table 2). In the three‐chamber social approach test used to assess sociability and social novelty preference in mice, the total distance traveled was significantly lower in OVX mice than in sham-operated controls, and locomotor activity was higher in *App*^*NL-G-F/NL-G-F*^ mice than in WT mice (Fig. 1H, J). In the sociability test, OVX WT mice showed no significant preference for the stranger mouse compared to the empty cage, whereas all other groups exhibited a significant preference (Fig. 1I). This observation suggests that OVX attenuated sociability-related behavior in WT mice, but not in *App*^*NL-G-F/NL-G-F*^ mice. In the social novelty preference test, sham-operated WT mice tended to show a preference for the stranger mouse compared to the familiar mouse, but OVX mice showed no significant preference for the stranger mouse (Fig. 1K). Sham-operated *App*^*NL-G-F/NL-G-F*^ mice showed a significant preference for the stranger mouse, while OVX *App*^*NL-G-F/NL-G-F*^ mice showed no significant preference for the stranger mouse (Fig. 1K). These results suggest that while OVX impaired sociability-related behavior in WT mice, the effect was smaller in *App*^*NL-G-F/NL-G-F*^ mice.

### ***OVX reversed the anxiolytic phenotype of App***^***NL-G-F/NL-G-F***^*** mice***

**Fig. 2:**
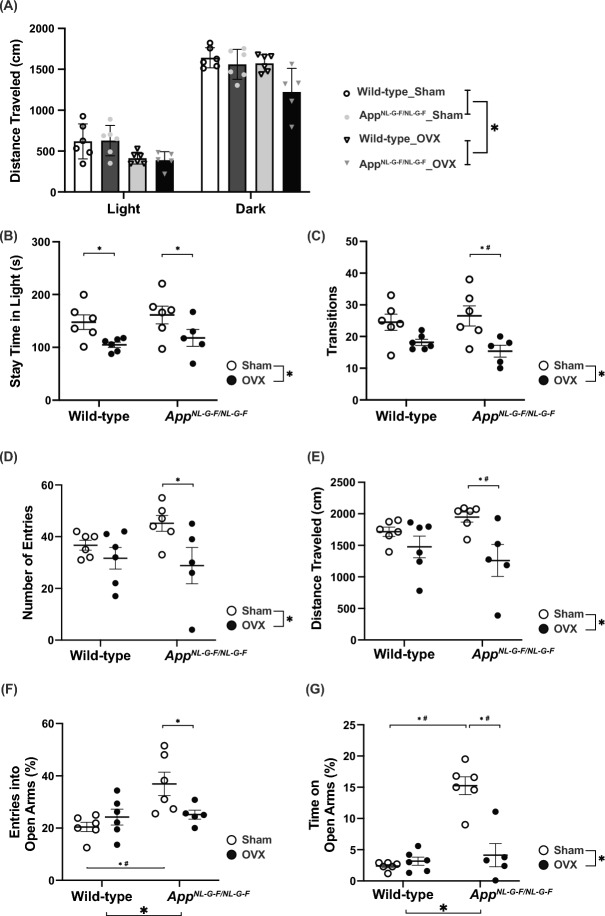
Ovariectomy (OVX) reversed anxiety-like behavior in *App*^*NL-G-F/NL-G-F*^ mice. In the light and dark transition test, distance traveled (**A**), time spent in the light chamber (**B**), and total number of light/dark transitions (**C**) are shown. In the elevated plus maze test, the number of arm entries (**D**), distance traveled (**E**), percentage of entries into open arms (**F**), and percentage of time on open arms (**G**) are shown. Data are presented as dot plot individual values and means ± S.E.M. (Sham, n = 6/wild-type or 6/*App*^*NL-G-F/NL-G-F*^; OVX, n = 6/wild-type or 5/*App*^*NL-G-F/NL-G-F*^). As in Fig. 1, *p < 0.05; #*p* < 0.0125 after Bonferroni correction.

We evaluated anxiety-like behaviors in OVX WT and *App*^*NL-G-F/NL-G-F*^ mice. In the light and dark transition test, OVX treatment decreased locomotor activity in both genotypes (treatment effect, *p* = 0.0013; genotype effect, *p* = 0.0634; treatment × genotype, *p* = 0.1948, Fig. 2A). In addition, the time spent in the light chamber was significantly reduced in OVX mice compared with sham-operated controls (treatment effect, *p* = 0.0052; genotype effect, *p* = 0.3442; treatment × genotype, *p* = 0.9798; Fig. 2B); the number of transitions between the chambers was also significantly lower in OVX mice (treatment effect, *p* = 0.0015; genotype effect, *p* = 0.8727; treatment × genotype, *p* = 0.3254; Fig. 2C). These results suggested that OVX reduced locomotor activity and increased anxiety-like behavior in both genotypes. In the elevated plus maze test, the total number of arm entries and distance traveled were decreased by OVX (total number of arm entries; treatment effect, *p* = 0.0195; genotype effect, *p* = 0.5091; treatment × genotype, *p* = 0.1904; Fig. 2D, distance traveled; treatment effect, *p* = 0.0067; genotype effect, *p* = 0.9512; treatment × genotype, *p* = 0.1585; Fig. 2E). *App*^*NL-G-F/NL-G-F*^ mice made a higher percentage of entries into the open arms and spent a significantly higher percentage of time on the open arms compared with WT mice (percentage of entries into the open arms; genotype effect, *p* = 0.0106; treatment effect, *p* = 0.2103; genotype × treatment, *p* = 0.0205; Fig. 2F, percentage of time on open arms; genotype effect, *p* < 0.0001; treatment effect, *p* = 0.0003; genotype × treatment, *p* < 0.0001; Fig. 2G). Compared with sham-operated *App*^*NL-G-F/NL-G-F*^ mice, however, OVX *App*^*NL-G-F/NL-G-F*^ mice made a lower percentage of entries into the open arms and spent a lower percentage of time on the open arms. These findings suggest that while anxiety-like behavior was decreased in *App*^*NL-G-F/NL-G-F*^ mice, OVX reversed this behavioral phenotype.

### Marginal effect of OVX on working memory task performance

**Fig. 3:**
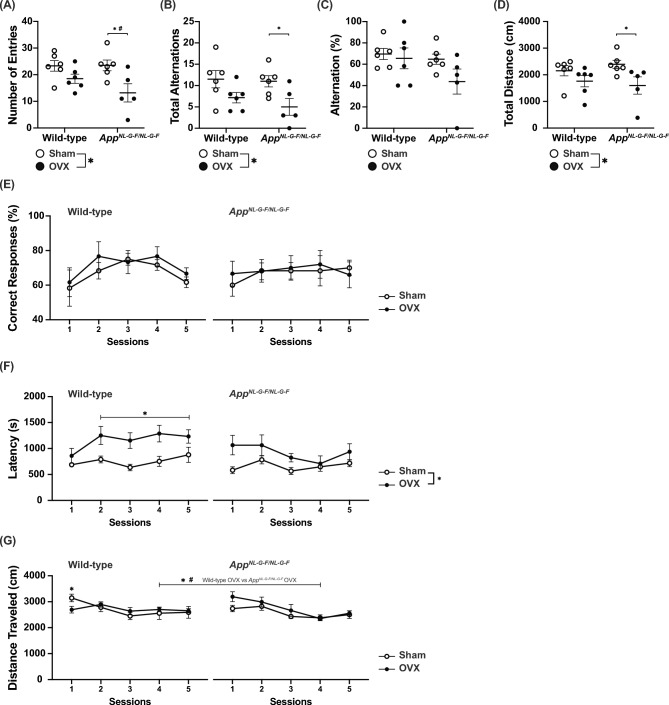
Ovariectomy (OVX) did not affect spatial memory task performance in *App*^*NL-G-F/NL-G-F*^ or wild-type (WT) mice. The spatial memory task performance of OVX *App*^*NL-G-F/NL-G-F*^ and WT mice. In the Y-maze test, the number of entries (**A**), total alternations (**B**), ratio of alternation (**C**), and distance traveled (**D**) are shown. Data are presented as dot plot individual values and means ± S.E.M. (Sham, n = 6/WT or 6/*App*^*NL-G-F/NL-G-F*^; OVX, n = 6/WT or 5/*App*^*NL-G-F/NL-G-F*^). In the T-maze test, the ratio of correct responses (**E**), latency (**F**), and distance traveled (**G**) are shown. Data are presented as means ± S.E.M. for the indicated numbers of animals (Sham, n = 6/WT or 6/*App*^*NL-G-F/NL-G-F*^; OVX, n = 6/WT or 5/*App*^*NL-G-F/NL-G-F*^). As in Fig. 1, *p < 0.05; #*p* < 0.0125 after Bonferroni correction.

The Y-maze and T-maze tests were used to evaluate cognitive function of OVX WT and *App*^*NL-G-F/NL-G-F*^ mice. In the Y-maze test, the number of entries into each arm, total alternations, and total distance traveled were significantly decreased in OVX mice compared to sham-operated controls (Fig. 3A, B, C). In contrast, no significant differences in alternation rates were observed over the 10-min test (Fig. 3D). In the T-maze spontaneous alternation test, the percentage of correct responses did not differ significantly among groups (Fig. 3E). OVX significantly increased the trial latency (treatment effect, *p* = 0.0097; genotype effect, *p* = 0.1956; treatment × genotype, *p* = 0.6126; Fig. 3F). OVX WT mice had longer trial latencies than sham-operated mice in trials 2 to 5, while the differences in latencies of *App*^*NL-G-F/NL-G-F*^ mice was not significant (WT mice; treatment effect, *p* = 0.0583; treatment × trial, *p* = 0.0327; trials 2–5; *p* = 0.0405; *App*^*NL-G-F/NL-G-F*^ mice; treatment effect, *p* = 0.0723; treatment × trial, *p* = 0.1176). With regard to the distance traveled, the interaction between genotypes, OVX, and the trial was significant (treatment effect, *p* = 0.4025; genotype effect, *p* = 0.6533; treatment × genotype, *p* = 0.4548; treatment × trial, *p* = 0.7453; genotype × trial, *p* = 0.2615; treatment × genotype × trial, *p* = 0.0143; Fig. 3G). OVX WT mice showed lower locomotor activity than sham-operated WT mice in the first trial (*p* = 0.0429). In the fourth trial, OVX *App*^*NL-G-F/NL-G-F*^ mice showed lower locomotor activity than OVX WT mice (*p* = 0.008). These results from the Y-maze and T-maze tests suggest that OVX did not affect the performance of spatial working memory in either genotype.

### OVX enhanced fear memory task performance

**Fig. 4:**
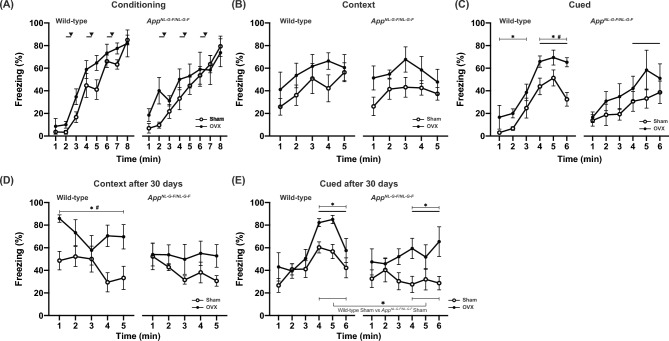
Ovariectomy (OVX) improved fear memory task performance in *App*^*NL-G-F/NL-G-F*^ and wild-type (WT) mice. The fear memory task performance of OVX *App*^*NL-G-F/NL-G-F*^ and WT mice. The percentage of freezing time in the conditioning (**A**), context testing (**B, D**), and cued testing with altered context (**C, E**) conditions are shown. Data are presented as means ± S.E.M. for the indicated numbers of animals. As in Fig. 1, *p < 0.05; #*p* < 0.0125 after Bonferroni correction.

We also examined fear memory in OVX mice using contextual and cued fear conditioning tests. In the conditioning period, freezing responses of all groups were similarly increased, with a significant interaction between genotype and time (treatment effect, *p* = 0.1078; genotype effect, *p* = 0.7245; treatment × genotype, *p* = 0.8817; treatment × time, *p* = 0.0722; genotype × time, *p* = 0.0016; treatment × genotype × time, *p* = 0.1716; Fig. 4A). In the context test at 24 h after conditioning, the effect of OVX on freezing was significant (treatment effect, *p* = 0.0166; genotype effect, *p* = 0.7285; treatment × genotype, *p* = 0.7764; treatment × time, *p* = 0.8376; genotype × time, *p* = 0.4747; treatment × genotype × time, *p* = 0.8896; Fig. 4B), although this effect did not remain significant after correction for multiple comparisons. In the cued test 24 h after conditioning, the effect of OVX was also significant in both pre-tone (genotype effect, *p* = 0.4288; treatment effect, *p* = 0.0344; genotype × treatment, *p* = 0.7438; genotype × time, *p* = 0.1767; treatment × time, *p* = 0.7346; genotype × treatment × time, *p* = 0.7452) and cued periods (genotype effect, *p* = 0.1338; treatment effect, *p* = 0.0168; genotype × treatment, *p* = 0.6191; genotype × time, *p* = 0.1631; treatment × time, *p* = 0.7147; genotype × treatment × time, *p* = 0.2043; Fig. 4C). OVX significantly increased the freezing ratio during the cued period in WT mice (treatment effect, *p* = 0.0052; treatment × trial, *p* = 0.276) but not in *App*^*NL-G-F/NL-G-F*^ mice (treatment effect, *p* = 0.2857; treatment × trial, *p* = 0.4838; Fig. 4C). Taken together, in the fear memory task conducted 24 h after conditioning, OVX improved the memory performance of WT mice. After 30 days, all mice were exposed to the same context and cue again. In the context test, the OVX effect on freezing remained significant (genotype effect, *p* = 0.0665; treatment effect, *p* = 0.001; genotype × treatment, *p* = 0.2012; genotype × time, *p* = 0.8836; treatment × time, *p* = 0.5571; genotype × treatment × time, *p* = 0.4141; Fig. 4D). OVX WT mice had a significantly higher freezing ratio than sham-operated WT (sham-operated WT vs OVX WT, *p* = 0.0047). OVX *App*^*NL-G-F/NL-G-F*^ mice also had a higher freezing ratio but the difference was not significant (sham-operated *App*^*NL-G-F/NL-G-F*^ vs OVX *App*^*NL-G-F/NL-G-F*^, *p* = 0.0995). In the cued period of the test, both genotype and OVX effects were significant (cued period; treatment effect, *p* = 0.0011; genotype effect, *p* = 0.0084; treatment × genotype, *p* = 0.5741; treatment × time, *p* = 0.9325; genotype × time, *p* = 0.0025; treatment × genotype × time, *p* = 0.169; Fig. 4E). OVX significantly increased the freezing response ratio in the cued period in both genotypes (sham-operated WT vs OVX WT, *p* = 0.0162; sham-operated *App*^*NL-G-F/NL-G-F*^ mice vs OVX *App*^*NL-G-F/NL-G-F*^ mice, *p* = 0.0278; Fig. 4E). In addition, sham-operated *App*^*NL-G-F/NL-G-F*^ mice had a lower freezing ratio compared with sham-operated WT mice (sham-operated WT vs sham-operated *App*^*NL-G-F/NL-G-F*^ mice, *p* = 0.028; Fig. 4E). OVX *App*^*NL-G-F/NL-G-F*^ mice had a lower freezing ratio than sham OVX WT during minutes 4 and 5 of the trial (OVX WT vs OVX *App*^*NL-G-F/NL-G-F*^ mice; genotype effect, *p* = 0.1394; genotype × trial, *p* = 0.0207; minutes 4 and 5 of the trial; genotype effect; *p* = 0.0144; genotype × trial, *p* = 0.1745). These results suggest that OVX increased long-term fear memory performance in mice and could improve cued long-term memory performance in *App*^*NL-G-F/NL-G-F*^ mice.

### Other behaviors

To comprehensively evaluate the behavioral phenotypes, we also conducted a hot plate test and startle response/prepulse inhibition test. No significant differences in the test results were detected between groups with respect to either the genotype or OVX treatment (Table 2).

### OVX reduced amyloid-β staining signal in WT mice

**Fig. 5:**
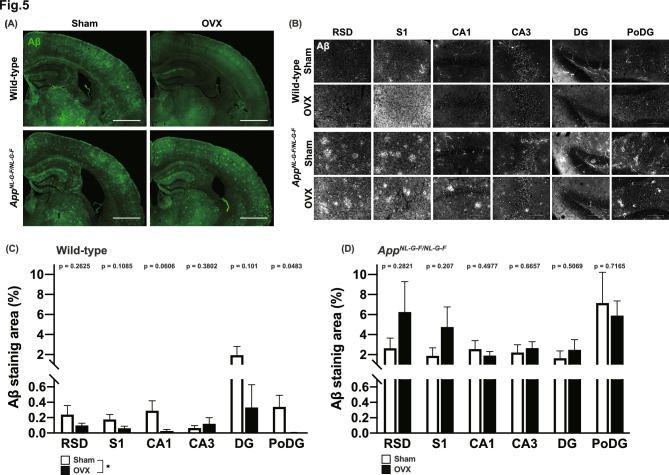
Ovariectomy (OVX) reduced amyloid-β staining in OVX wild-type (WT) mice. OVX-related changes in amyloid-β deposition. Representative images of each genotype, treatment, and brain area are shown (**A, B**). Scale bars = 1 mm (**A**), 100 μm (**B**). Expression of amyloid-β was decreased in OVX WT mice but not in OVX *App*^*NL-G-F/NL-G-F*^ mice (**C, D**). Data are presented as means ± S.E.M. for the indicated numbers of animals. As in Fig. 1, *p < 0.05. The P values indicate a treatment effect in one‐way ANOVA for each brain region. RSD, retrosplenial dysgranular cortex; S1, somatosensory cortex; CA, central amygdaloid nucleus; DG, dentate gyrus; PoDG, polymorph layer of the dentate gyrus.

To investigate the effects of OVX at the brain tissue level, we performed amyloid-β staining in both groups. In both genotypes, amyloid-β plaque formation was observed (Fig. 5A). *App*^*NL-G-F/NL-G-F*^ mice showed stronger amyloid-β staining than WT mice (Fig. 5A, B). Interestingly, OVX WT mice showed reduced amyloid-β staining compared to sham-operated controls (Fig. 5C), but no such changes were observed in *App*^*NL-G-F/NL-G-F*^ mice (Fig. 5D). These results suggest that OVX attenuates amyloid plaque formation, a pathologic phenotype of AD, although this effect was limited to WT mice.

## Discussion

AD poses a greater risk in women than in men^2–4^, but the actual relationship between AD and the factors specific to women, such as hormonal changes, menopause, and lifespan, remain unclear. Given the long-term progressive nature of AD, early prevention and treatment strategies, as well as models that consider sex differences, are critically needed. Therefore, in the present study, we conducted a comprehensive behavioral test battery to investigate the effect of OVX in our preclinical AD model mice. Our findings revealed that *App*^*NL-G-F/NL-G-F*^ mice exhibited reduced anxiety-like behavior and a non-significant trend toward impaired contextual memory. Those behavioral phenotypes are consistent with the previously reported phenotypes of *App*^*NL-G-F/NL-G-F*^ male and female mice^28–31^. Interestingly, however, OVX recovered these behavioral phenotypes of *App*^*NL-G-F/NL-G-F*^ mice to WT levels. In addition, our findings indicate that the mutation in *App*^*NL-G-F/NL-G-F*^ mice may play a protective role in OVX-induced phenotypes, such as body weight gain, impaired motor coordination, and reduced sociability. Therefore, the results of our study suggest that the preclinical AD female mouse model exhibits an AD-like behavioral phenotype and OVX partially attenuates the AD-like phenotype under certain conditions.

In humans, menopause is associated with weight gain and negative affective states, which are also observed in OVX animal models^20,32–39^. These findings are consistent with our results, in which OVX WT mice exhibited weight gain, reduced activity, and impaired motor coordination. AD models combined with OVX treatment have shown similar outcomes; for example, previous studies using female triple transgenic AD (3 × TgAD) mice reported that the reduction in estrogen due to OVX impairs metabolism, resulting in increased body weight^40^. These phenotypes, however, were not observed in *App*^*NL-G-F/NL-G-F*^ mice. In this study, the behavioral phenotypes observed in *App*^*NL-G-F/NL-G-F*^ mice were largely consistent with previous reports using male mice^29,30^. Nevertheless, although previous studies reported impaired working memory in *App*^*NL-G-F/NL-G-F*^ mice^28,29^, we detected no significant differences in working memory performance between *App*^*NL-G-F/NL-G-F*^ and WT mice in tasks such as the Y-maze and T-maze. This discrepancy may be attributed to differences in the age of the mice used. While the previous studies primarily used male mice and conducted tests at 3–6 months of age^28,29^, we used female mice tested at around 10 months of age. Notably, performance in the T-maze task is reported to decline even in WT mice by 12 months of age^41^, s suggesting that age-related impairments in the WT group may have reduced the observable differences between genotypes. Moreover, certain behavioral phenotypes observed in other AD models were not evident in *App*^*NL-G-F/NL-G-F*^ mice. For example, APPswe/PS male mice, a transgenic AD model, exhibit deficits in both sociability and social memory^42^. In contrast, we found no significant difference in sociability and social memory did not significantly differ between *App*^*NL-G-F/NL-G-F*^ mice and WT mice. In addition, OVX decreased sociability in WT mice, but not in *App*^*NL-G-F/NL-G-F*^ mice. These differences could be due to several factors, including the sex of the mice used in the study. One possible reason for the absence of certain behavioral phenotypes observed in other AD models is the difference in the promoter used. While our model utilizes an endogenous *App* promoter to express amyloid-β protein^28^, the other transgenic models utilized different exogenous promoters^43,44^, which may result in a different spatiotemporal accumulation of amyloid plaques or neuronal damage in the brain. Indeed, previous studies reported that amyloid plaque deposition is milder in *App*^*NL-G-F/NL-G-F*^ mice compared to other transgenic AD models^45^. Thus, as a preclinical model, *App*^*NL-G-F/NL-G-F*^ mice may exhibit subtler symptoms and a distinct pattern of amyloid pathology, which could explain the absence of certain behavioral phenotypes such as deficits in sociability. Furthermore, our results suggest that *App*^*NL-G-F/NL-G-F*^ mice have low susceptibility to some of the OVX-induced behavioral phenotypes. One possible explanation is that, although this model represents a preclinical stage of AD, neuropathologic alterations may have already occurred by the age at which OVX was performed, resulting in a blunted response to the treatment. For example, glial fibrillary acidic protein (GFAP), a marker of astrocyte activation and neuroinflammation, is upregulated around 6 months of age in A*pp*^*NL-G-F/NL-G-F*^ mice^30^. It is also known that GFAP expression can increase with age and following OVX^46,47^. Therefore, in *App*^*NL-G-F/NL-G-F*^ mice, neural inflammation may already be elevated due to AD-like pathology, irrespective of OVX treatment, potentially reducing the observable effects of OVX when compared to WT mice. The mechanisms underlying the resistance of *App*^*NL-G-F/NL-G-F*^ mice to these phenotypes remain unclear. Our findings highlight the need for further cross-model analyses across diverse AD models.

Surprisingly, OVX normalized anxiety-like behavior and memory impairment of our preclinical AD model to WT levels. The combination of the OVX and AD model, including genetically engineered mice, has often been used to explore sex differences in AD pathology^17,18,24,48–50^. In these models, OVX mainly caused exacerbation of AD-related phenotypes^17,48,49^, and these phenotypes were sometimes attenuated by hormone supplementation^24,40,48^. OVX can induce anxiety-like behavior in rodent models^20–22^. These findings are consistent with our results, revealing that OVX restored the reduced anxiety-like behavior of *App*^*NL-G-F/NL-G-F*^ mice to the levels observed in WT mice. In contrast, few reports have demonstrated a positive effect of OVX on memory performance^14–16,51^. Interestingly, the present study showed that OVX also enhanced long-term memory in WT mice. In rat and monkey, OVX protects against the development of aging-related spatial memory deficits^14–16,51^. Consistent with our findings, a recent report demonstrated that OVX treatment enhanced fear conditioning performance in *App*^*NL-G-F/NL-G-F*^ mice, although WT data were not included^52^. While we interpret the persistent freezing as evidence of long-term retention, it is also possible that the results reflect impaired or incomplete extinction processes. In either case, these findings suggest that certain OVX-induced mechanisms facilitate the formation of fear memory. Although OVX appeared to facilitate fear memory formation, such phenomena were not observed in working memory tasks such as the T-maze and Y-maze. As discussed earlier, this may be due to the relatively advanced age of the mice used in the present study, making it difficult to detect changes in tasks that rely heavily on spontaneous activity. Alternatively, it is important to note that fear conditioning tasks assess associative memory involving the amygdala, hippocampus, and prefrontal cortex^53,54^, whereas Y-maze and T-maze tasks primarily reflect spatial working memory dependent on the hippocampus^55,56^. Thus, the observed OVX-induced enhancement of fear memory may reflect a selective modulation of circuits distinct from those involved in spatial working memory. The results of some human clinical studies indicate OVX generally increases the risk of dementia and AD^57–62^, while others suggest that OVX increases the risk of dementia only when performed prematurely^63,64^. In our study, OVX was performed at approximately 4.5 months of age, which corresponds to the young adult stage according to the age classification by Flurkey et al.^65^. We chose this timing to specifically examine the effects of the loss of fully mature ovarian function in a preclinical AD model. Behavioral testing was conducted during a period corresponding to the transition from the end of young adulthood to middle age in mice. During this period, even in sham-operated WT mice, ovarian function naturally becomes less stable. Indeed, although not directly related to behavioral phenotypes, previous studies reported that the effects of OVX can vary depending on the timing of the procedure^66^. These observations underscore the complexity of OVX effects in AD models and highlight the importance of considering age and disease stage at the time of the intervention. It is therefore possible that the effects observed in the present study may not occur if OVX is performed at an earlier stage. Taken together, these results suggest a complex interaction between OVX and AD-related genotypes, with time-dependent effects of ovarian hormone withdrawal.

In the C57BL/6 J strain used in the present study, endogenous amyloid-β is reported to form plaques as animals age^67^. Interestingly, our data showed that OVX in WT mice tended to attenuate amyloid-β staining, particularly in the hippocampal region. The hippocampus is a brain area critically involved in several behavioral phenotypes observed in this study, including anxiety-like behavior and fear conditioning^53,68–70^. While no attenuation of Aβ plaques was observed in OVX *App*^*NL-G-F/NL-G-F*^ mice, it is possible that OVX reduces age-related amyloid deposition and attenuates the AD-like behavioral phenotype. These data indicate that OVX may be protective against AD-like symptoms under certain conditions, although the underlying mechanisms require further investigation. Furthermore, our results suggest that our mouse model may be useful for investigating the prevention and amelioration of clinical symptoms of menopause-associated AD.

In the present study, we investigated the impact of OVX on various behaviors in a preclinical AD model. Our findings suggest that ovarian hormone withdrawal due to OVX influences behavioral and pathologic phenotypes in a complex manner. While estrogen depletion is often associated with exacerbation of AD symptoms, our results indicate that OVX may also trigger compensatory mechanisms that mitigate certain AD-related phenotypes. Potential factors contributing to this effect could include changes in neuroinflammatory responses, neurotrophic factor levels, or metabolic adaptations following ovarian hormone loss^71–73^. While OVX is commonly used as a model of menopause or perimenopause, it results in an abrupt cessation of ovarian hormone production. In this study, we utilized the OVX model to investigate the role of ovarian function in a preclinical AD model. To fully understand the impact of physiologic, gradual ovarian changes on AD pathology, however, future studies will need to complement this approach with other menopause models. Moreover, although OVX is specific to female models, the underlying mechanisms of this protective effect may not be exclusive to females. It is highly plausible that this protective effect is based on shared physiologic pathways or factors in both sexes. Demetriou et al. demonstrated in the same preclinical model that OVX enhanced fear conditioning memory, and that administration of an ERβ agonist further amplified this effect; notably, a similar enhancement was also observed in male mice^52^. While this might appear to reflect compensation for the loss of ovarian function due to OVX, it is important to note that endogenous ERβ agonists can also be produced by non-ovarian sources such as the adrenal glands^74–76^. Together with our findings in WT mice, this suggests that endogenous ERβ ligands derived from non-ovarian sources may contribute to the attenuation of AD-related pathology. Further studies are needed to elucidate the molecular and cellular mechanisms underlying these protective effects and to determine whether similar mechanisms exist in males. By clarifying the underlying mechanisms, our model could be useful for investigating potential strategies to ameliorate AD pathology.

## Materials and methods

### Animals and experimental design

In this study, WT and *App*^*NL-G-F/NL-G-F*^ mice^28^ were generated by in vitro fertilization and embryo transfer of heterozygotes’ sperm and oocytes. The heterozygous mice were maintained on a C57BL/6 J background purchased from Charles River Laboratories Japan (Yokohama, Japan). The mice were group‐housed (3 to 4/cage) in individually ventilated cages (IVC; 19.7 × 34.0 × 16.5 cm) in a room maintained at 24 ± 3˚C with a 12‐h light/dark cycle (lights on at 7:00 am) and ad libitum access to food and water. Bilateral ovariectomy surgical manipulations were conducted under appropriate anesthesia (a combination anesthetic 0.3 mg/kg of medetomidine, 4.0 mg/kg of midazolam, and 5.0 mg/kg of butorphanol) at the age of 18 weeks (sham-operated WT, n = 6; OVX WT, n = 6; sham-operated App^NL-G-F/NL-G-F^, n = 6; OVX *App*^*NL-G-F/NL-G-F*^, n = 5). Their behaviors were assessed with a battery of behavioral tests starting at 35 weeks of age.

### Behavioral tests

The mice were subjected to a battery of behavioral tests in the following sequence: general health and neurologic screening (body weight, body temperature, and grip strength), the light/dark transition, open field, hot plate, social interaction, elevated plus maze, rotarod, startle response/prepulse inhibition, Y-maze, T-maze spontaneous alternation, three‐chamber social approach and contextual/cued fear conditioning test. The schedule of comprehensive test battery of this study is exhibited in Table1. All of mice went through the same test battery on the same day so that they experienced all of the behavioral tests in the same order. After each test, the floors and walls of the testing apparatuses were cleaned with 70% ethanol solution or super hypochlorous water to prevent a bias caused by olfactory cues. The behavioral tests were performed between 8:30 am and 6:00 pm. Information about each mouse and the behavioral data collected in this study are available in the “Mouse Phenotype Database” (http://www.mouse-phenotype.org/).

#### Neurological screen and neuromuscular strength test (GHNS)

The righting, whiskers twitch, and ear twitch reflexes were evaluated. A number of physical features, including the presence of whiskers or bald hair patches, were also recorded. Body weight and rectal temperature were measured. Neuromuscular strength was assessed ng the grip strength and wire hang tests. A grip strength meter (O’Hara & Co., Tokyo Japan) was used to assess forelimb grip strength. Mice were lifted and held by their tail so that their forepaws grasp a wire grid. The mice were then gently pulled backward by the tail until they released the grid. The peak force applied by the forelimbs of the mouse was recorded in Newtons (N). Each mouse was tested three times, and the largest value was used for statistical analysis.

#### Light/dark transition test (LD)

The light/dark transition test, developed by Crawley and colleagues^77^, was performed as previously described^78^. The apparatus comprised a cage (21 × 42 × 25 cm) divided into two sections of equal size divided by a partition with a door (O’Hara & Co., Tokyo, Japan). One chamber was brightly illuminated (380 ± 20 lx), whereas the other was dark (0.2 ± 0.1 lx). Mice were placed into the dark chamber and were allowed to move freely between the two chambers for 10 min with the door open. The distance traveled (cm), the total number of transitions between compartments, latency to first enter the light chamber (s), and time spent in the light chamber (s) were recorded automatically using the ImageLD program.

#### Open field test (OF)

Locomotor activity was measured using an open field test. Each mouse was placed in the corner of the open field apparatus (40 × 40 × 30 cm: AccuScan Instruments, Columbus, OH, USA). The center of the floor was illuminated at 100 lx. Total distance traveled (cm), vertical activity (rearing measured by counting the number of photobeam interruptions), time spent in the center area (20 × 20 cm), and beam‐break counts for stereotypic behaviors were recorded. Data were collected for a period of 120 min.

#### Hot plate test (HP)

The hot plate test was used to evaluate sensitivity to a painful stimulus. The mice were placed on a 55.0 (± 0.3) ˚C hot plate (Columbus Instruments, Columbus, OH, USA), and the latency to the first hind-paw response was recorded. The hind-paw response was defined as either a foot shake or a paw lick.

#### Social interaction test (SI)

The social interaction test was conducted to measure social behavior in a novel environment, as previously described^79^. Weight‐matched (within 9.3 g) mice of the same treatment group that had been housed in different cages were placed together into an acrylic box (40 × 40 × 30 cm) and allowed to explore freely for 10 min. The total number of contacts, total duration of contacts (s), total duration of active contacts (s), mean duration per contact (s), and total distance traveled (cm) were recorded and analyzed automatically using the ImageSI program. Active contact was defined as the two mice contacted each other and one or both mice moved with a velocity of at least 10 cm/s.

#### Elevated plus maze test (EP)

The elevated plus maze test, which is widely used to assess anxiety-like behavior, was performed as previously described^80^. The apparatus comprised two arms without walls (open arms, 25 × 5 cm), two arms of the same size with 15‐cm‐high transparent walls (closed arms), and a central square (5 × 5 cm) connecting the arms, which were at 90˚ to each other (O’Hara & Co.). The arms and central square were made of white plastic plates and were elevated to a height of 55 cm above the floor. The open arms were surrounded by a raised ledge (3 mm thick and 3 mm high) to prevent mice from falling off the open arms. Arms of the same type were located opposite one another. Each mouse was placed in the central square of the maze facing one of the closed arms. The number of arm entries, distance traveled (cm), percentage of entries into the open arms, and percentage of time spent in the open arms were measured during a 10‐min test period. Data acquisition and analysis were performed automatically using the ImageEP program.

#### Rotarod test (RR)

Motor coordination and balance were tested with the rotarod test. The rotarod test, using an accelerating rotarod (UGO Basile Accelerating Rotarod, Varese, Italy), was performed by placing the mice on a rotating drum (3 cm diameter) and measuring the time each animal maintained its balance on the drum. The speed of the rotarod accelerated from 4 to 40 rpm over a 5-min period. Each mouse underwent three consecutive trials per day for two consecutive days (total of six trials).

#### Startle response/prepulse inhibition test (PPI)

The startle response and prepulse inhibition test were performed as previously described^81^. A startle reflex measurement system (O’Hara & Co.) was used. The mice were placed in a Plexiglas cylinder and left undisturbed for 10 min. The test comprised two test trials with the startle stimulus only and four test trials for prepulse inhibition. White noise (40 ms) was used as the startle stimulus for all trials. The startle response was recorded for 140 ms (measuring the response every 1 ms) starting with the onset of the prepulse stimulus. The background noise level in each chamber was 70 dB. The peak startle amplitude recorded during the 140-ms sampling window was used as the dependent variable. The intensity of the startle stimulus was 110 or 120 dB. The prepulse sound was presented 100 ms before the startle stimulus, and its intensity was 74 or 78 dB. Four combinations of prepulse and startle stimuli were employed (74–110, 78–110, 74–120, and 78–120 dB). The mean inter-trial interval was 15 s (range 10–20 s).

#### Y-maze test

Y-maze test was performed as previously described^82^. Exploratory activity was measured using a Y-maze apparatus (arm length: 40 cm, arm bottom width: 3 cm, arm upper width: 10 cm, height of wall: 12 cm). Each subject was placed in the center of the Y-maze field. The numbers of entries and alterations were recorded using a modified version of the ImageYM program. Data were collected for 10 min.

#### T-maze test

T-maze spontaneous alternation tests was performed as previously described^83^ using the automatic modified T-maze apparatus (O’Hara & Co., Tokyo, Japan). In the spontaneous alternation task, mice were allowed to freely run for 10 laps in a session and subjected to 5 sessions in total. A correct response in each lap indicated that mice chose the opposite direction from the last lap. Data acquisition and analysis were performed automatically using ImageTM.

#### Three‐chamber social approach test (TCSI)

The three‐chamber social approach test is used to investigate sociability and preference for social novelty in mice^84^. The apparatus comprised a rectangular, three‐chambered box and a lid with a video camera (O’Hara & Co., Tokyo, Japan). Each chamber was 20 × 40 × 47 cm, and the dividing walls were made from clear Plexiglas with a small square opening (5 × 3 cm) allowing access into each chamber. The tests were performed as previously described^84^, with a slight modification as follows: subject mice were placed in the three‐chambered box and allowed to explore for 10 min before the sociability test was conducted (habituation session), and during the session, empty wire cages (9 cm in diameter, 11 cm in height, with vertical bars 0.5 cm apart) were located in the corner of each outside compartment. During the following session, an unfamiliar C57BL/6 J male mouse (stranger 1) that had had no prior contact with the subject mouse was placed into a wire cage located in one of the side chambers. The location of the stranger mouse in the left vs right chamber was systematically alternated between trials. The subject mouse was placed in the central compartment and allowed to explore the entire box for 10‐min to assess sociability (sociability test). Next, a second stranger male mouse was placed into the wire cage in the other outside compartment that had been empty during the first 10‐min session to evaluate social preference for a new stranger (social novelty preference test). Thus, the subject mouse had a choice between the first, already‐investigated, now‐familiar mouse (stranger 1) and the novel unfamiliar mouse (stranger 2). The amount of time spent in each chamber and time spent around each cage were automatically calculated from video images using the ImageCSI program.

#### Contextual and cued fear conditioning test and extinction test (FZ)

The fear conditioning test was conducted using an automated video‐analysis system as previously described^85^. Mice were placed in a conditioning chamber (26 × 34 × 29 cm) in a sound‐attenuated room and allowed to explore freely for 2 min. The animals were presented with an auditory cue (55 dB white noise) that served as a conditioned stimulus (CS) for 30 s. During the last 2 s of the CS, mice were given a footshock (0.3 mA, 2 s) as an unconditioned stimulus (US). Two more CS‐US pairings were presented at 120‐s intervals. One day and 30 days after the conditioning session, a context test was performed in the conditioning chamber. A cued test in an altered context was performed after the context test using a triangular box (35 × 35 × 40 cm) made of white opaque plastic, which was located in a different sound‐attenuated room. In the cued test, after the initial 3‐min period of no CS presentation, the CS was presented during the last 3‐min period of the test.

Freezing during each minute of the test was measured automatically using the ImageFZ program in the same manner as previously described.

## Data analysis in behavioral tests

Behavioral data were obtained automatically by applications (ImageLD^78^, ImageSI^79^, ImageEP^80^, ImageTM^83^, ImageCSI^86^, ImageYM^82^, and ImageFZ^85^) based on the public domain NIH Image program and ImageJ program, and modified for each test. The plugins are freely available on the “Mouse Phenotype Database” website (http://www.mouse-phenotype.org/software.html)^87^.

## Immunohistochemistry

Mice were administered a supralethal dose of pentobarbital sodium (100 mg/kg, i.p.) and then perfused with phosphate-buffered saline (PBS) and 4% paraformaldehyde (PFA) in 0.1 M sodium phosphate buffer, pH 7.4. The brains were dissected, immersed overnight in 4% PFA in 0.1 M sodium phosphate, and transferred to 30% sucrose in PBS for at least 3 days for cryoprotection. The brain samples were mounted in 33% Tissue-Tek® O.C.T. Compound (4583, Sakura Finetek USA, Inc.) diluted by 30% sucrose in PBS, frozen, and cut into 40-μm-thick coronal sections using a microtome (CM3050 S, Leica, Nussloch, Germany). For antigen retrieval, the sections were incubated in 90% formic acid for 5 min at room temperature. The sections were then incubated at 4 °C overnight with primary antibody specific to Aβ (4G8, Covance, 1:1000 dilution). To detect antigen localization, the sections were incubated at 4 °C overnight with Alexa Fluor® 488 AffiniPure Donkey Anti-Mouse IgG (H + L) (715–545-151, Jackson Immunoresearch, 1: 1000 dilution; Invitrogen). Stained sections were observed using an immunofluorescence microscope, BZ-X710 (Keyence, Osaka, Japan). For statistical analysis, the data from two sections per mouse from 3 individual mice in each group were used.

## Statistical analysis

Statistical analyses were performed using StatView for Windows version 5.0.1, (SAS Institute, Cary, NC, USA). Data were analyzed using one-way or two-way ANOVA followed by Fisher’s LSD test, two-way repeated ANOVA, paired t-test or Kruskal–Wallis test where appropriate. Values in graphs are presented as mean ± S.E.M. The post-hoc multiple comparisons were further performed using Fisher’s PLSD with Bonferroni correction. The results of the statistical analysis of comprehensive behavioral analysis described in Table 2.

## Data Availability

Raw data on the behavioral test and information about each mouse are accessible on the public database “Mouse Phenotype Database” (http://www.mouse-phenotype.org/).

## Declarations

### Animal studies

Animal care and experimental protocols were approved by the Animal and Gene Experiment Committee of the University of Toyama (Authorization No. G2016OPR-3, G2021OPR-1, A2016OPR-3, A2019OPR-2 and A2022OPR-2) and conducted in accordance with the Guidelines for the Care and Use of Laboratory Animals of the University of Toyama and the ARRIVE guidelines (https://arriveguidelines.org).
